# The role of mitochondrial fusion protein 2 in regulating ER stress in premature placental aging: A case-control study

**DOI:** 10.1097/MD.0000000000050212

**Published:** 2026-08-14

**Authors:** Dandan Sun, Ling Ai, Ping Xu, Jiawei Zhou, Lili Xue

**Affiliations:** a Jiaxing Women and Children Hospital Affiliated to Wenzhou Medical University, Jiaxing, Zhejiang, China; b Women and Children Hospital Affiliated to Jiaxing University, Jiaxing, Zhejiang, China; c Department of Obstetrics, Jiaxing Municipal Maternal and Child Health Care Hospital, Jiaxing, Zhejiang, China.

**Keywords:** advanced maternal age, endoplasmic reticulum stress, mitochondrial fusion protein 2, placental senescence, protein kinase R-like ER kinase

## Abstract

Advanced maternal age (AMA) is associated with an increased risk of adverse perinatal outcomes, which may be attributed to premature placental aging. Previous studies have demonstrated that mitochondrial fusion protein 2 (Mfn2) plays a critical role in aging-related neurodegenerative disorders, metabolic diseases, and vascular pathologies. However, its expression in AMA pregnancies and its impact on placental function remain poorly understood. This study aimed to investigate the expression patterns of Mfn2 in placental tissues from AMA pregnancies and elucidate its regulatory role in endoplasmic reticulum (ER) stress through the protein kinase R-like ER kinase (PERK) signaling pathway. The downregulation of Mfn2 expression in AMA exacerbates placental senescence by dysregulating ER stress via PERK. Mfn2 binds to PERK, inhibits phosphorylation, and alleviates oxidative stress and senescence, highlighting its therapeutic potential. Placental tissues from 20 women with AMA and 20 young women were analyzed using real-time quantitative reverse transcription PCR‌. An H_2_O_2_-induced HTR-8/SVneo trophoblast cell line senescence model was established. Functional assays (cell counting kit-8, wound healing, Transwell, and β-galactosidase staining) and oxidative stress markers (reactive oxygen species/malondialdehyde/superoxide dismutase) were evaluated. Western blotting was used to assess the expression of PERK pathway proteins (phosphorylated protein kinase R-like endoplasmic reticulum kinase, activating transcription factor 4, and CCAAT/enhancer binding protein homologous protein) and senescence markers (P16 and P53). Immunoprecipitation verified the Mfn2-PERK interaction. Mfn2 expression was significantly downregulated in AMA placentas and negatively correlated with PERK expression. Mfn2 overexpression reversed the H_2_O_2_-induced suppression of trophoblast viability, migration, invasion, and senescence while reducing the levels of reactive oxygen species and malondialdehyde and restoring the activity of superoxide dismutase. Mfn2 inhibited PERK pathway activation (phosphorylated protein kinase R-like endoplasmic reticulum kinase, activating transcription factor 4, and CCAAT/enhancer binding protein homologous protein) and the expression of senescence markers (P16/P53). Mfn2 interacted directly with PERK.

## 1. Introduction

In recent years, the number of pregnant women of advanced maternal age (AMA) has increased.^[1]^ AMA is a serious threat to the life and health of pregnant women and is among the main causes of various perinatal and long-term complications in offspring.^[2]^ Epidemiological studies have shown that pregnant women of advanced age are more likely to have adverse perinatal outcomes; however, the specific mechanism is unclear and may be caused by premature placental aging.

Premature placental aging is associated with many adverse pregnancy outcomes, including fetal growth restriction (FGR), preeclampsia, spontaneous preterm birth, and intrauterine fetal death. Placentas with FGR show age-related phenotypes, including telomere shortening, telomere aggregation or dysfunction, and reduced telomerase activity.^[3]^ Compared with that in women with normal blood pressure, the formation of telomere aggregates (senescence-associated heterochromatic punctas) in preeclamptic pregnancies is increased, and the expression of the senescence-inducing factors tumor protein p53 (P53), cyclin-dependent kinase inhibitor 1A (P21), and cyclin-dependent kinase inhibitor 2A (P16) is increased.^[4]^ Spontaneous premature birth is closely associated with placental aging.^[5]^ Aging has both favorable and unfavorable effects on pregnant tissues, depending on the type of cell senescence and duration of aging. A deep understanding of the physiological and pathological mechanisms of placental aging will help us correctly understand the physiological functions of the placenta and help treat and prevent pregnancy-related diseases.

Recent research has shown that ER stress (ERS) may also play an important role in placental aging in advanced pregnancy.^[6]^ There are many ERS markers, such as advanced glycation end products, CCAAT/enhancer binding protein homologous protein (CHOP), and X-box binding protein 1, in placental tissues during advanced pregnancy. In addition, the levels of ERS-related proteins in the placental tissues of advanced pregnancy significantly increase. Sierra et al^[7]^ reported that the endoplasmic reticulum (ER) can also accelerate aging because ERS can lead to decreased cell function and apoptosis. The accumulation of erroneous and unfolded proteins activates the ERS response, which leads to apoptosis, and a moderate response ultimately reduces damage and protects cells, but at the cost of a loss of cell function. The cell membrane and secretory proteins need to be processed and mature in the ER. Owing to translation inhibition, protein synthesis decreases, which inevitably affects the maturation and secretion of membrane proteins and secretory proteins, causing a decline in the function of the corresponding tissues. If this stress response lasts for a short time, the decline in tissue function can be recovered quickly. In most cases, the ERS response does not last long; however, aging is a continuous process. During the aging process, various types of damage frequently occur, causing chronic ERS.^[8]^ Chronic ERS is inevitably accompanied by continuous and cumulative damage to tissue and organ function, that is, aging. Research has also shown that ERS is involved not only in the pathogenesis of a variety of geriatric diseases but also in the aging process itself.^[8,9]^

Mitochondrial fusion protein 2 (Mfn2) is a transmembrane motor protein located in the outer mitochondrial membrane. It is a key factor in regulating mitochondrial fusion and maintaining mitochondrial structure.^[10]^ Studies^[11,12]^ have shown that the expression level of Mfn2 is closely related to ERS. Under ERS, the expression level of Mfn2 is significantly reduced, thereby affecting the metabolism and physiological functions of cells. Previous studies^[13]^ have shown that Mfn2 plays an important role in regulating placental ERS in preeclampsia. However, whether it can mediate placental aging by regulating ERS has not yet been reported. In this study, we detected the expression of Mfn2 in the placentas of older women and constructed a trophoblast senescence cell model to determine whether Mfn2 inhibits cell senescence by binding to protein kinase R-like ER kinase (PERK) to inhibit its phosphorylation and regulate ERS. These results suggest that Mfn2 may be a potential therapeutic target for the treatment of premature placental aging.

## 2. Materials and methods

### 2.1. Subjects

Term placental tissue from women who underwent young pregnancies (20–25 years old; 37–41 weeks; n = 20) or AMA pregnancies (35–45 years old; 37–41 weeks; n = 20) was collected from Jiaxing Municipal Maternal and Child Health Care Hospital. Patients with pregnancy complications such as gestational diabetes mellitus, FGR, spontaneous abortion, renal disease, or preeclampsia were excluded. Immediately after vaginal delivery or cesarean section, each placenta was placed on a sterile tray on ice, and from each placenta, 4 full-thickness biopsies (approximately 1 cm × 1 cm × 1 cm each) were obtained from the central area of the maternal side, avoiding the basal plate and the cord insertion site; the biopsies were taken from the villous parenchyma after removing the decidual layer. Each biopsy was rinsed 3 times with ice-cold sterile phosphate-buffered saline (PBS, pH 7.4) to remove excess blood, then immediately snap-frozen in liquid nitrogen and stored at −80 °C until further analysis, and all specimens were pathologically confirmed by hematoxylin and eosin staining to ensure representative placental villous tissue without infarction or infection. All participants signed an informed consent form, and the study was approved by the Ethics Committee of Jiaxing Municipal Maternal and Child Health Care Hospital affiliated with Jiaxing University.

### 2.2. Reverse transcription quantitative polymerase chain reaction

Total ribonucleic acid was extracted from the cells using TRIzol reagent (Invitrogen) according to the manufacturer’s protocol. To detect Mfn2 and PERK expression, the PrimeScript II 1st Strand complementary deoxyribonucleic acid Synthesis Kit (Takara Bio Inc.) was used to produce complementary deoxyribonucleic acid, and quantitative polymerase chain reaction was performed using the SYBR Premix Ex Taq kit on an Applied Biosystems 7500 thermocycler. β-actin was used as an internal reference. Relative expression levels were computed using the 2^−ΔΔCT^ method. Sequence-specific primers were synthesized as follows:

Mfn2 sense: 5′-ATCTGTGCCAGCAAGTTGACA-3′;

Mfn2 antisense: 5′-AAGTGAATCCAGAGCCTCGAC-3′;

PERK sense: 5′-TCATCCAGCCTTAGCAAACC-3′;

PERK antisense: 5′-ATGCTTTCACGGTCTTGGTC-3′;

β-actin sense: 5′-ATTGCCGACAGGATGCAGAA-3′;

β-actin antisense: 5′-GCTGATCCACATCTGCTGGAA-3′.

### 2.3. Cell culture

The HTR-8/SVneo cell line was obtained from the Chinese Academy of Sciences (Shanghai, China). HTR-8/SVneo cells were cultured in RPMI-1640 medium (Sigma–Aldrich) supplemented with 10% fetal bovine serum (FBS; Thermo Fisher Scientific, Inc.), 100 IU/ml penicillin, and 100 IU/ml streptomycin (both from Thermo Fisher Scientific, Inc.) at 37 °C in a humidified atmosphere containing 5% CO_2_. After the cells adhered and grew to 80% confluence, the culture medium was removed, the cells were washed with an appropriate amount of PBS, and the cells were digested with 0.25% trypsin (Sigma–Aldrich; Merck KGaA). Cells in the logarithmic growth phase were selected for subsequent experiments.

### 2.4. Expression constructs and cell transfection

The sequence for FLAG-tagged Mfn2 was amplified by PCR and subcloned into the pCMV-Tag2 vector (Stratagene). The constructs were verified by deoxyribonucleic acid sequencing. Transient cell transfections were performed using the standard calcium phosphate method. An appropriate number of cells were seeded into 6-well plates and grown to approximately 80% confluence. Transfection was performed using Lipofectamine 3000 (Invitrogen, L3000015) transfection reagent according to the manufacturer’s instructions.

### 2.5. H_2_O_2_ treatment

The transfected HTR-8/SVneo cells were washed with PBS and treated with 100 μM H_2_O_2_ (hydrogen peroxide) in the culture medium. After being cultured for another 48 hours, the cells were subjected to subsequent experiments.

### 2.6. Western blotting

Total protein was extracted according to the manufacturer’s instructions, and the protein concentrations were measured using the bicinchoninic acid method. The proteins were subjected to 12% sodium dodecyl sulfate–polyacrylamide gel electrophoresis and transferred to a polyvinylidene fluoride membrane. The membranes were blocked with 5% skim milk at room temperature for 2 hours and then washed with Tris-buffered saline with Tween 20. The membranes were incubated overnight at 4°C with anti-Mfn2 (Abcam, ab124773), anti-CHOP (Abcam, abab11419), anti-PERK (Abcam, ab229912), anti-FLAG (Huabio, 0912-1), and anti-β-actin (ab8226; Abcam) primary antibodies. The primary antibodies were diluted to 1:500 in Tris-buffered saline with Tween 20. After being washed, the membranes were incubated with secondary antibodies for 1 hour at 37°C. An enhanced chemiluminescence kit was used to visualize the protein bands. β-actin was used as a loading control, as described in our previous study.^[13]^

### 2.7. Cell viability assay

We used a cell counting kit-8 (Dojindo) to examine cell viability according to the manufacturer’s instructions. Approximately 1 × 10^4^ HTR-8/SVneo cells were seeded into 96-well culture plates. At 24, 48, or 72 hours after transfection, the cells were incubated with 100 µL of water-soluble tetrazolium salt-8 reagent at 37°C for 4 hours. The absorbance of the cells at 450 nm (OD450) was subsequently measured.

### 2.8. Wound healing assay

HTR-8/SVneo cells were cultured in 6-well plates at a density of 1 × 10^5^ cells per well. After 24 hours of culture, the cells were transfected using Lipofectamine 3000. After 12 hours, wounds (1 mm wide) were created using plastic scribers. The scratch wounds were photographed over a 72-hour period using a microscope (CX31; Olympus Corporation).

### 2.9. Transwell assay

Transwell experiments were performed according to the manufacturer’s protocol to evaluate cell migration and invasion abilities. Briefly, 1 × 10^5^ HTR-8/SVneo cells were prepared in serum-free RPMI-1640 medium and added to the upper chamber of a Transwell plate. For the invasion assay, the upper chambers were coated with Matrigel (BD Biosciences), whereas the Matrigel coating was omitted for the migration experiment. Afterwards, a total of 500 μL of RPMI-1640 medium supplemented with 10% fetal bovine serum was added to the lower chamber for the HTR-8/SVneo cells. After incubation for 24 hours, the cells were fixed with paraformaldehyde, stained with 0.1% crystal violet, and counted under a microscope (CX31; Olympus Corporation).

### 2.10. Measurement of reactive oxygen species (ROS)

HTR-8/SVneo cells were seeded in 6-well plates and washed 3 times with PBS after various treatments. Afterwards, 2′-7′-dichlorodihydrofluorescein diacetate (CA1410; Solarbio), one of the most widely used reagents to directly measure intracellular ROS levels, was added to the plates, which were then incubated for 20 minutes at 37°C in the dark. Finally, green fluorescence indicating the level of intracellular ROS was observed by fluorescence microscopy (Olympus) at 100 × magnification, and the mean fluorescence intensity was calculated using ImageJ software to determine the level of intracellular ROS.

### 2.11. Detection of superoxide dismutase (SOD) levels

The levels of SOD in the harvested protein supernatants were evaluated using the SOD detection kit protocol provided by the manufacturer (Elabscience Biotechnology Co., Ltd.).

### 2.12. Detection of malondialdehyde (MDA) levels

The levels of MDA in the harvested protein supernatants were evaluated by enzyme-linked immunosorbent assays (Elabscience Biotechnology Co., Ltd., Wuhan, China) following the MDA detection kit protocol provided by the manufacturer.

### 2.13. Statistical analysis

GraphPad Prism 8 software (La Jolla, CA, USA) was used for statistical analyses. Statistical data are expressed as the mean ± standard deviation. Student *t* test was used to compare groups, and statistical significance was set at *P* < .05.

### 2.14. Mfn2 is frequently downregulated in AMA tissues and is negatively correlated with the expression of the ERS sensor PERK

**Figure 1.**
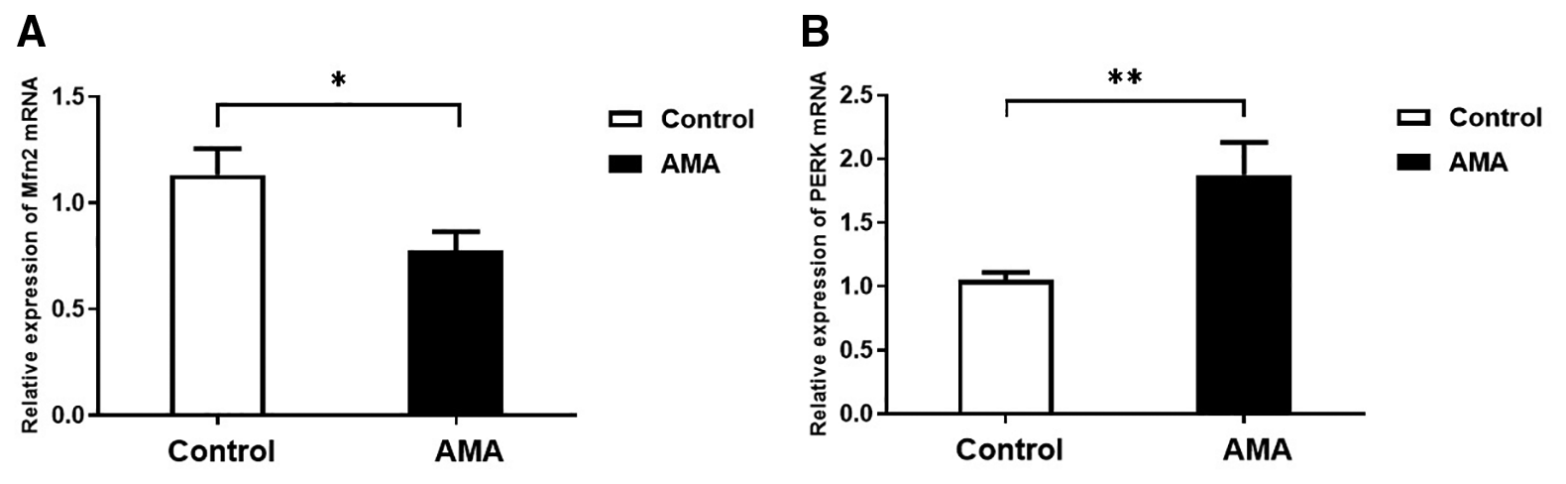
The expression of Mfn2 and PERK in young pregnant mothers (control group) and advanced pregnant mothers (AMA group) was measured by qRT–PCR. (A) qRT–PCR analysis of Mfn2 and (B) PERK expression in AMA. * *P *< .05, * *P* < .01 vs control. AMA = advanced maternal age, Mfn2 = mitochondrial fusion protein 2, mRNA = messenger ribonucleic acid, PERK = protein kinase R-like ER kinase, qRT–PCR = real-time quantitative reverse transcription PCR‌.

The expression of Mfn2 in 20 women with young maternal age pregnancies (control group) and 20 women with AMA pregnancies (AMA group) was measured by real-time quantitative reverse transcription PCR‌. The results revealed that the expression of Mfn2 was lower in AMA tissues than in normal tissues (Fig. 1A). In contrast, PERK expression was significantly upregulated (Fig. 1B). Correlation analysis revealed that in the placental tissues of older pregnant women, the expression of these 2 genes was negatively correlated (*r* = −0.5583, *P* < .05), indicating that Mfn2 may play an inhibitory role in AMA.

### 2.15. Mfn2 overexpression reverses H_2_O_2_-induced decreases in trophoblast cell viability, migration, invasion, and aging

**Figure 2.**
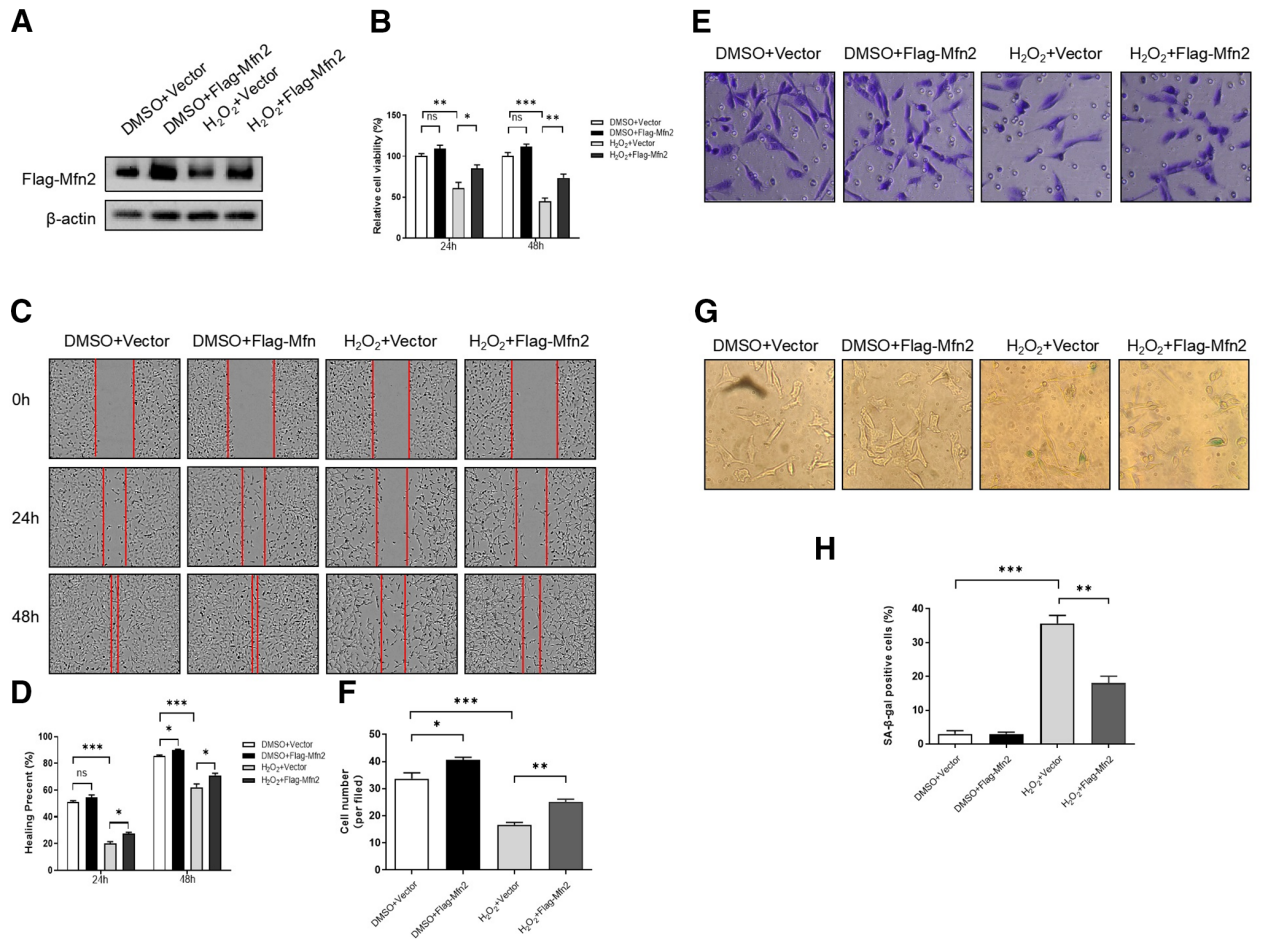
Mfn2 overexpression reverses H_2_O_2_-induced decreases in trophoblast cell viability, migration, invasion, and aging. (A) The levels of Mfn2 were assessed in H_2_O_2_-induced senescent HTR-8/SVneo cells after transfection with FLAG-Mfn2 or vector by Western blotting; (B) CCK-8 assays; (C and D) wound healing assays; (E and F) Transwell assays; and (G and H) β-galactosidase staining were performed to determine whether the overexpression of Mfn2 reversed the H_2_O_2_-induced inhibition of trophoblast cell viability, migration, invasion, and aging. * *P* < .05, ** *P* < .01, *** *P* < .001 vs control. CCK-8 = cell counting kit-8, DMSO = dimethyl sulfoxide, H_2_O_2_ = hydrogen peroxide, Mfn2 = mitochondrial fusion protein 2.

To investigate the functional role of Mfn2 in trophoblast cells, we transfected HTR-8/SVneo cells with FLAG-tagged Mfn2 and established an H_2_O_2_-induced trophoblast (HTR-8/SVneo) senescent cell model. The expression levels of Mfn2 were significantly upregulated in cells transfected with FLAG-tagged Mfn2 compared with those in the control vector group after 48 hours (Fig. 2A). A cell counting kit-8 assay revealed that the upregulation of Mfn2 reversed the H_2_O_2_-induced decrease in trophoblast cell viability (Fig. 2B), whereas wound healing and Transwell assays revealed that the upregulation of Mfn2 also strongly reversed the H_2_O_2_-induced inhibition of cell migration (Figs. 2C and 2D) and invasion (Figs. 2E and 2F). In addition, we tested the effect of Mfn2 on H_2_O_2_-induced trophoblast senescence. As shown in Figure 2G and 2H, the results of *β*-galactosidase staining revealed that the upregulation of Mfn2 reversed H_2_O_2_-induced trophoblast cell senescence. These results suggest that the overexpression of Mfn2 reverses H_2_O_2_-induced decreases in trophoblast cell viability, migration, invasion, and aging.

### 2.16. Mfn2 inhibits H_2_O_2_-induced ERS in trophoblast cells

**Figure 3.**
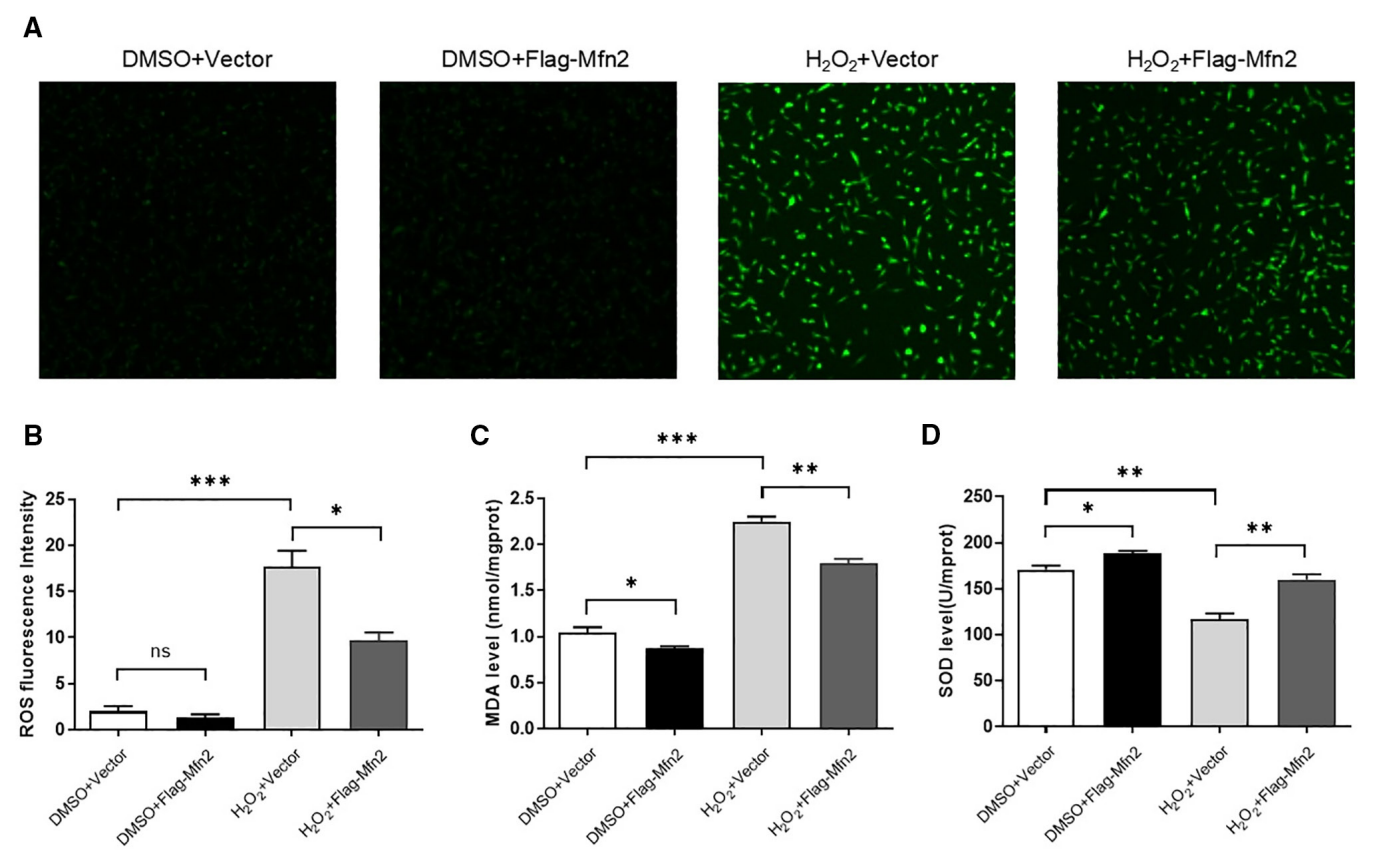
Mfn2 inhibits H_2_O_2_induced ERS in trophoblast cells. (A and B) ROS; (C) MDA; and (D) SOD levels were detected using DCFH-DA, MDA, and SOD assays, respectively, in an H_2_O_2_-induced senescent HTR-8/SVneo cell model after transfection with FLAG-Mfn2 or vector. * *P *< .05, ** *P *< .01, *** *P *< .001 vs control. DCFH-DA = 2′-7′-dichlorodihydrofluorescein diacetate, ERS = endoplasmic reticulum stress/ER stress, H_2_O_2_ = hydrogen peroxide, MDA = malondialdehyde, Mfn2 = mitochondrial fusion protein 2, ROS = reactive oxygen species, SOD = superoxide dismutase.

Recent studies have shown that ERS may also play an important role in placental aging in advanced pregnancy. To verify whether Mfn2 inhibits trophoblast cell senescence and is related to ERS, we tested the inhibitory effect of Mfn2 overexpression on H_2_O_2_-induced ERS in trophoblast cells. As shown in Figure 3A and 3B, the immunofluorescence results revealed that the overexpression of Mfn2 attenuated the level of ROS induced by H_2_O_2_ in trophoblast cells (*P* < .05). Moreover, the results of the MDA and SOD tests revealed that the overexpression of Mfn2 reversed the increase in MDA (*P* < .01) and the decrease in SOD (*P* < .01) induced by H_2_O_2_ (Figs. 3C and 3D). These results suggest that Mfn2 inhibits H_2_O_2_-induced ERS in trophoblasts.

### 2.17. Mfn2 inhibits trophoblast cell senescence by regulating ERS

**Figure 4.**
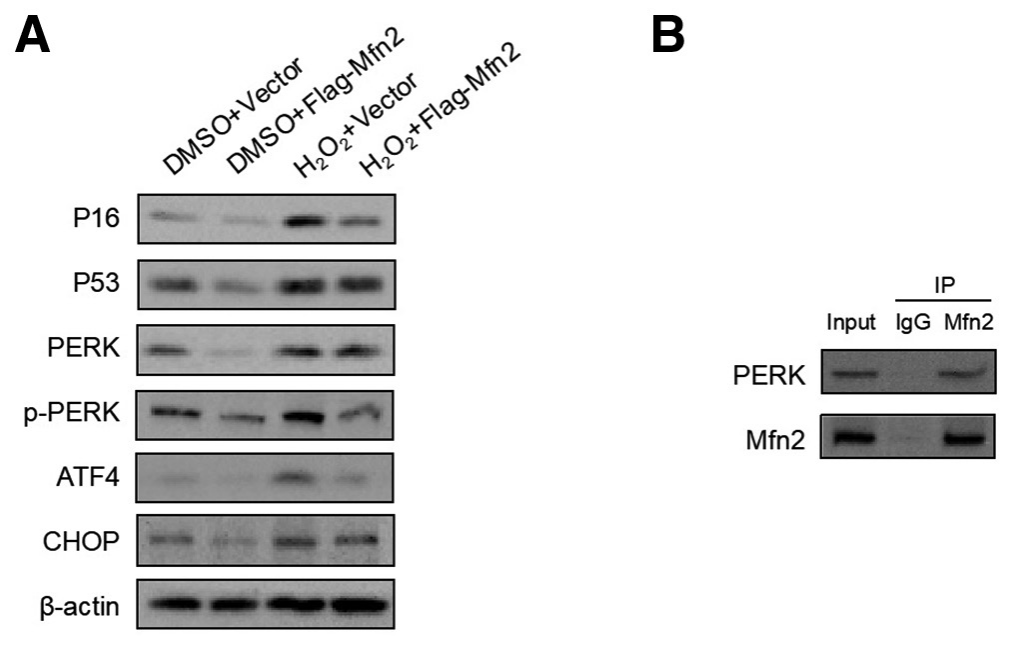
Mfn2 inhibits trophoblast cell senescence by regulating ERS. (A) The levels of PERK, ATF4, CHOP, and PERK phosphorylation in the PERK signaling pathway and the expression of the cell senescence markers P16 and P53 were assessed in an H_2_O_2_-induced senescent HTR-8/SVneo cell model after transfection with FLAG-Mfn2 or vector by Western blotting; (B) Immunoprecipitation assay for detecting the interactions between endogenous Mfn2 and PERK in trophoblast cells. ATF4 = activating transcription factor 4, CHOP = CCAAT/enhancer binding protein homologous protein, ERS = endoplasmic reticulum stress/ER stress, H_2_O_2_ = hydrogen peroxide, IgG = immunoglobulin G, MDA = malondialdehyde, Mfn2 = mitochondrial fusion protein 2, PERK = protein kinase R-like ER kinase.

We further verified whether Mfn2 inhibits the senescence of trophoblast cells by regulating the PERK signaling pathway during ERS. The expression of PERK signaling pathway proteins involved in ERS following Mfn2 overexpression in a cell model of H_2_O_2_-induced trophoblast cell senescence was detected. As shown in Figure 4A, Mfn2 overexpression downregulated the expression of PERK, activating transcription factor 4, and CHOP, as well as the expression of the cell senescence markers P16 and P53. The results of the immunoprecipitation assay revealed that Mfn2 interacts with PERK in trophoblast cells (Fig. 4B). These results indicate that Mfn2 alleviates trophoblast cell senescence by interacting with PERK and inhibiting PERK phosphorylation and ERS in trophoblast cells.

## 3. Discussion

Physiological aging of the placenta is an inevitable biological process.^[14]^ As a temporary organ of the human body, the placenta grows and develops rapidly with increasing gestational age, and the need for oxygen and nutrition increases exponentially. This causes the placenta to operate at a high load in late pregnancy, and placental villus trophoblast cells are subjected to stress. Changes in placental villus structure eventually lead to aging and dysfunction of the placenta, thus affecting fetal development and the life safety of pregnant women.^[7,9]^ However, owing to the compensatory mechanism of the human body, the normal aging process of the placenta is a physiological phenomenon that does not affect the safety of the mother or fetus. Abnormal or premature placental aging interferes with the normal physiological function of the placenta, especially its nutrient–oxygen exchange function. Such abnormalities may lead to adverse pregnancy outcomes. If the placenta shows an obvious aging phenotype during early pregnancy, the load of the placenta begins to increase. When the load of the placenta reaches its peak in late pregnancy and it becomes difficult to meet normal nutrient exchange at the maternal–fetal interface, it severely affects the safety of the mother and fetus.^[15]^ Studies have reported that the occurrence and development of premature birth, FGR, stillbirth, and preeclampsia are related to abnormal placental aging.^[3,16]^ The placental aging in advanced pregnancy investigated in this study has an adverse effect on placental function. AMA is a serious threat to the life and health of pregnant women and is among the main causes of perinatal and long-term complications.

Studies have shown that regulating the state of ERS in an organism can directly or indirectly affect its lifespan, and the level of ERS also changes significantly with aging.^[17–19]^ Moreover, factors related to aging, such as oxidative stress, also function to activate ERS. They can promote the aging of organisms by promoting ERS-mediated cell dysfunction and apoptosis. Thus, ERS is closely associated with aging. However, the understanding of the connection between placental aging and ERS is still in its infancy, and the relevant molecular mechanisms remain unclear.

The ER is an organelle wrapped by a biological membrane in the cytoplasm and is involved mainly in biological processes such as protein synthesis, modification, and folding.^[20]^ ERS refers to the state of cell stress that occurs when the ER loses balance. ERS is manifested mainly through ER expansion, protein aggregation, and protein glycosylation, and these abnormalities trigger a series of cell stress response mechanisms. Studies have shown that ERS is closely associated with aging.^[21]^ ERS can affect apoptosis by regulating the expression of B-cell lymphoma 2 family members, thereby leading to aging.^[21]^ ERS can also affect oxidative stress by regulating the expression of antioxidant enzymes, thereby leading to organ dysfunction and aging. In addition, ERS can also participate in the regulation of inflammatory responses by regulating the expression of inflammatory factors.^[22]^ Furthermore, ERS can lead to cell cycle disorders and cell proliferation blockade, further affecting the development, function, and aging of the body.^[23]^ The role of ERS in the promotion of placental aging may be related to this mechanism.

Mfn2 is an important mitochondrial fusion regulatory protein that participates in the regulation of mitochondrial dynamics.^[10]^ The regulatory roles of mitochondrial dynamics include mitochondrial fusion and fission. Mitochondrial fusion refers to the merging of 2 adjacent mitochondria, whereas mitochondrial fission refers to the fission of 1 mitochondrion into 2. Mitochondrial fusion can be divided into the fusion of the outer and inner mitochondrial membranes. The Mfn located on the outer membrane of mitochondria regulates the fusion process of the outer membrane of mitochondria, and Mfn can be divided into 2 subtypes: Mfn1 and Mfn2.^[24]^ Mfn2 participates in the fusion of mitochondrial and ER membranes, maintaining the stability of cellular energy metabolism and calcium ion balance. Studies have shown that the expression level of Mfn2 is closely related to ERS.^[12]^ Under ERS, the expression level of Mfn2 is significantly reduced, thereby affecting the metabolism and physiological functions of cells. Mfn2 also participates in apoptosis induced by ERS. Mfn2 inhibits ERS-induced cell death by maintaining the stability of mitochondrial function.^[25]^ In addition, a study revealed that Mfn2 can affect the occurrence and development of inflammatory responses by inhibiting the activation of the nuclear factor-κB signaling pathway, reducing the production and release of inflammatory factors, and regulating the production and clearance of mitochondrial ROS.^[26]^ These results suggest that Mfn2 is an important regulatory molecule of ERS.

However, when ERS occurs, 3 downstream signal transduction proteins related to the unfolded protein response can be activated (PERK, inositol-requiring kinase, and activating transcription factor 6) which can change the transcription and translation levels of specific target genes and reduce the number of unfolded proteins in cells, thereby maintaining normal physiological function. When persistent or severe ERS occurs, these 3 signaling molecules can activate downstream apoptotic signaling pathways, such as the CHOP (growth arrest and deoxyribonucleic acid damage-inducible gene 153) and cysteine-containing aspartate proteinase 12, to induce apoptosis. Among the 3 signaling pathways that lead to apoptosis due to ERS, the PERK pathway is the most important.^[27]^ In this study, we detected and correlated the expression of Mfn2 and PERK in the placental tissues of older women. The results revealed that Mfn2 expression was negatively correlated with PERK expression in the placental tissues of older women. Overexpression of Mfn2 in trophoblasts inhibits H_2_O_2_-induced trophoblast cell senescence. Immunoprecipitation experiments revealed that Mfn2 interacts with PERK in trophoblast cells and regulates the activation of the ERS-related PERK signaling pathway by inhibiting PERK phosphorylation. Mfn2 plays important roles in premature placental senescence by binding to PERK, inhibiting its phosphorylation, regulating ERS, and inhibiting cell senescence. Thus, Mfn2 may be a potential target for the prevention and treatment of premature placental senescence.

Several limitations of this study should be acknowledged. First, the sample size is relatively small (n = 20 per group). While the observed differences between groups reached statistical significance, the small sample size limits the statistical power to detect small effect sizes and may increase the risk of type II error (false negative) for secondary or exploratory analyses. Moreover, a small sample size may also lead to an overestimation of effect sizes (type I error risk) in the context of multiple comparisons. Therefore, the positive findings, particularly the correlation between Mfn2 and PERK expression, should be validated in larger, independent cohorts. Second, the limited sample size restricts the generalizability of our conclusions to broader populations, including different ethnic groups or women with other pregnancy complications. Third, the cross-sectional, case-control design identifies associations but is not designed to establish causality; therefore, prospective studies are needed to determine the causal relationship between Mfn2 downregulation and premature placental aging. Future prospective studies with larger sample sizes and longitudinal follow-up are needed to further confirm the mechanistic role of Mfn2 in regulating ERS and to assess its potential as a therapeutic target. Notwithstanding these limitations, the present study provides preliminary evidence supporting the involvement of the Mfn2-PERK axis in premature placental aging associated with AMA.

## 4. Conclusion

In summary, the downregulation of Mfn2 expression in AMA exacerbates placental senescence by dysregulating ERS via PERK. Mfn2 binds to PERK, inhibits phosphorylation, and alleviates oxidative stress and senescence, highlighting its therapeutic potential.

## Author contributions

**Conceptualization:** Lili Xue.

**Data curation:** Dandan Sun, Ling Ai, Ping Xu, Jiawei Zhou.

**Formal analysis:** Jiawei Zhou.

**Funding acquisition:** Dandan Sun.

**Investigation:** Ling Ai, Ping Xu.

**Methodology:** Ping Xu.

**Project administration:** Lili Xue.

**Resources:** Lili Xue.

**Software:** Ling Ai, Ping Xu.

**Supervision:** Lili Xue.

**Validation:** Ling Ai, Ping Xu.

**Writing – original draft:** Dandan Sun.

**Writing – review & editing:** Lili Xue.
